# Seeking Health Information Online: Association With Young Australian Women’s Physical, Mental, and Reproductive Health

**DOI:** 10.2196/jmir.4048

**Published:** 2015-05-18

**Authors:** Ingrid Jean Rowlands, Deborah Loxton, Annette Dobson, Gita Devi Mishra

**Affiliations:** ^1^Centre for Longitudinal and Life Course ResearchSchool of Public HealthUniversity of QueenslandBrisbaneAustralia; ^2^Priority Research Centre for Gender, Health and AgeingFaculty of Health and MedicineUniversity of NewcastleNewcastleAustralia

**Keywords:** Internet, women’s health, young adults, health status, mental health, information seeking behavior

## Abstract

**Background:**

Relatively little is known about the extent to which young adults use the Internet as a health information resource and whether there are factors that distinguish between those who do and do not go online for health information.

**Objective:**

The aim was to identify the sociodemographic, physical, mental, and reproductive health factors associated with young women’s use of the Internet for health information.

**Methods:**

We used data from 17,069 young women aged 18-23 years who participated in the Australian Longitudinal Study on Women’s Health. Multivariable logistic regression was used to estimate the association between sociodemographic, physical, mental, and reproductive health factors associated with searching the Internet for health information.

**Results:**

Overall, 43.54% (7433/17,069) of women used the Internet for health information. Women who used the Internet had higher odds of regular urinary or bowel symptoms (OR 1.44, 95% CI 1.36-1.54), psychological distress (very high distress: OR 1.24, 95% CI 1.13-1.37), self-reported mental health diagnoses (OR 1.16, 95% CI 1.09-1.23), and menstrual symptoms (OR 1.25, 95% CI 1.15-1.36) than women who did not use the Internet for health information. Internet users were less likely to have had blood pressure checks (OR 0.85, 95% CI 0.78-0.93) and skin cancer checks (OR 0.90, 95% CI 0.84-0.97) and to have had a live birth (OR 0.74, 95% CI 0.64-0.86) or pregnancy loss (OR 0.88, 95% CI 0.79-0.98) than non-Internet users.

**Conclusions:**

Women experiencing “stigmatized” conditions or symptoms were more likely to search the Internet for health information. The Internet may be an acceptable resource that offers “anonymized” information or support to young women and this has important implications for health service providers and public health policy.

##  Introduction

The affordability and availability of the Internet make it a convenient resource that is increasingly used to offer information, support, and services to the population regarding their health. Recent estimates from the United States and Europe suggest that almost half of adults seek health information online [1-3], often before or after a visit to a health care professional to obtain further information or advice [2,4,5]. Certain subgroups appear more likely to access health information online, including younger adults, women, and those from higher socioeconomic backgrounds [1,4,6-9]. However, few studies have examined the characteristics of online health seekers beyond sociodemographic factors.

Going online for health information may be useful for a broad range of health issues. The Internet offers diversity in health information and support with numerous websites, blogs, and online support groups all dedicated to various aspects of health. Of the few studies examining the health status of those who search the Internet for health information, those experiencing socially embarrassing or “stigmatizing” symptoms or conditions (eg, urinary incontinence and mental health conditions) [10], those wanting sexual health information (eg, sexually transmitted infections) [11], and pregnant women and mothers [9,12] appear to be more likely to seek health information online. However, studies specifically focusing on online health-seeking behaviors among young adults are limited. A recent population-based study from France reported that young women aged 15-30 years who had children or who were psychologically distressed were more likely to seek health information online [1]. However, the health care needs of adolescents and young adults are likely to be diverse and there may be better insights offered by research that targets specific age groups [13].

In this paper, we describe the health information sources used by a national sample of young Australian women aged 18-23 years. We aim to identify the sociodemographic, physical, mental, and reproductive health factors associated with searching the Internet for health information to inform health care services and support for young women.

## Methods

### Overview

The Australian Longitudinal Study on Women’s Health (ALSWH) is a national study focusing on the biological, psychological, social, and economic factors relevant to women’s health [14]. Initially, ALSWH used mailed self-report surveys to explore the health and well-being of 3 cohorts of Australian women aged 18-23 years, 45-50 years, and 70-75 years when the project began in 1996. The 40,000 participants were randomly selected using the national health insurance database (Medicare), which includes all permanent residents of Australia. Since 1998, surveys have been conducted on a triennial basis [14]. Comparisons with Australian census data show that the 3 cohorts of women are broadly representative of the Australian population in these age groups [15].

In 2012-2013, ALSWH recruited a new cohort of young women born 1989-1995 and aged 18-23 years when they were first surveyed. Women were eligible if they lived in Australia, had a valid Medicare number, and if they consented to data linkage (linking survey data with administrative health data). Approval for the study was obtained from the Human Research Ethics Committee of the University of Newcastle and the University of Queensland, as well as the Department of Human Services and the Department of Health. Further details of the survey methodology are available from the study website [16].

### Recruitment

Participants were recruited from October 2012 to December 2013 through conventional (ie, radio interviews and magazine advertising) and online social media (including YouTube videos), with full details reported elsewhere [17,18]. A total of 17,069 women completed a Web-based survey comprising 62 questions on sociodemographic characteristics (eg, educational qualifications), physical and mental health (eg, self-rated general health), anthropometric data (eg, height, weight), reproductive health (eg, pregnancy, birth outcomes), health behaviors (eg, physical activity levels, tobacco and illicit drug use), and experience of violence or abuse and access to health services (eg, screening services). Comparisons with national census data (2011) show that the 1989-1995 cohort is broadly representative of the Australian population of women aged 18-23 years, but with a slight overrepresentation of better-educated, Australian-born, and nonsmoking women [17].

### Study Variables

#### Outcome Measure

A question asking women, “Where do you get information about your health? (mark all that apply),” was used to categorize women into those who did and did not use the Internet as a source of health information. Women chose from 10 information sources (eg, Internet, family, doctor, television/radio/ magazines/posters/leaflet, other) and those who reported using the Internet (solely or in conjunction with other sources) were classified as “Internet users” and the remaining women were classified as “non-Internet users.” We also calculated the number of health information sources used by summing together women’s responses to the list of 10 sources (yes=1; no=0), creating an ordinal variable ranging from 0-10.

#### Sociodemographic Variables

We collected information on age (in years), area of residence based on an index of distance to the nearest urban center (major cities, inner regional, outer regional, remote/very remote) [19], highest level of education (less than year 12, year 12 or equivalent, certificate/diploma, university degree), current relationship status (never married, never married but in a relationship, married/engaged, separated/divorced/widowed), ability to manage on income (easy, not too bad, difficult some of the time, difficult all of time, impossible), and living arrangements (living with parents / not living with parents).

#### Health and Health Conditions

Women were asked to rate their general health (excellent, very good, good, fair or poor) and to report chronic health conditions (eg, diabetes, heart disease, cancer). Women reporting urinary/bowel symptoms (eg, urine that burns or stings, leaking urine, hemorrhoids, constipation), mental health conditions (eg, depression, anxiety, other), and who used preventative health services (eg, blood pressure or skin cancer checks in the last two years) were classified as “yes” or “no.”.

#### Sexual and Reproductive Health

Women reported if they ever had a live birth, pregnancy loss (ectopic pregnancy, miscarriage, termination for medical or personal reasons, stillbirth), sexually transmitted infection (chlamydia, genital herpes, genital warts, human immunodeficiency virus [HIV] / acquired immune deficiency syndrome [AIDS], hepatitis B/C), or received a diagnosis of endometriosis or polycystic ovary syndrome, or had a Papanicolaou test in the last 2 years (yes/no). Women reporting menstrual symptoms “sometimes” or “often” in the last 12 months (eg, vaginal discharge, heavy periods, severe period pain) were categorized as suffering these symptoms regularly and classified as “yes” vs “no.”

### Statistical Analysis

Bivariate logistic regression was used to estimate odds ratios (ORs) and 95% confidence intervals (95% CIs) for the association between sociodemographic, physical, mental, and reproductive health factors and searching the Internet for health information. Sociodemographic variables were entered into a multivariable logistic regression model to examine their association with Internet use for health information. The ORs for the association between physical, mental, and reproductive health factors and Internet use, adjusted for key sociodemographic characteristics, were estimated by multivariable logistic regression models. Data analysis was conducted using SAS version 9.4 (TS1M0) for Windows (SAS Institute Inc, Cary, NC, USA).

## Results

On average, women aged 18-23 accessed 3 sources of information for their health. Doctors (77.01%, 13,145/17,069) followed by family members (61.87%, 10,561/17,069) were the major sources of health information. The Internet and friends were identified by 43.55% (7433/17,069) and 43.25% (7383/17,069) of women, respectively, followed by school, university, and Technical and Further Education (TAFE; 39.55%, 6750/17,069), conventional media (32.18%, 5495/17,069; includes television, radio, magazines, posters, leaflets), and to a lesser extent, nurses (14.49%, 2474/17,069). A minority of women (5.90%, 1007/17,069) reported other sources of health information (results not shown).

**Figure 1:**
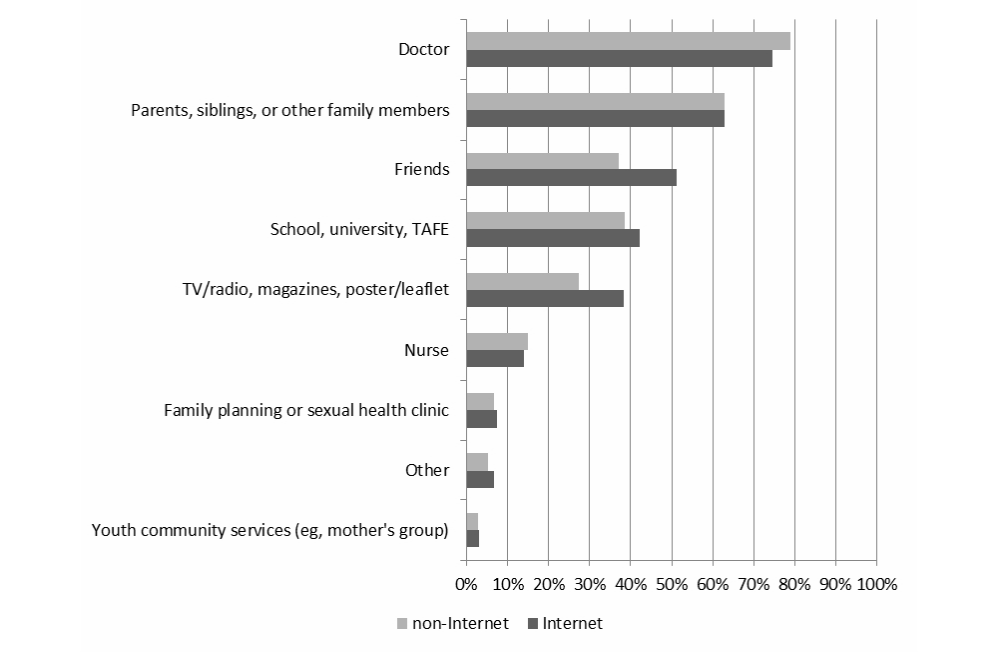
Sources of health information accessed by young women who do and do not use the Internet as a health information resource.

Overall 43.55% (7433/17,069) of women identified the Internet as a source of health information (either alone or in conjunction with other sources) with the remaining 56.45% (9636/17,069) of women using non-Internet sources only. Stratifying by Internet use made little difference to the overall pattern of health sources accessed (Figure 1). However, Internet users were more likely to rely on friends, school, university, and TAFE or conventional media than non-Internet users, whereas non-Internet users sought advice more often from doctors.

Being older, having a university education, living in a major city, being in a relationship (never married), and not living with parents were significantly associated with using the Internet for health information (Table 1). Income management was not associated with accessing the Internet for health information. After adjusting for sociodemographics, having urinary or bowel symptoms, moderate or higher levels of psychological distress, a diagnosed mental health condition, or menstrual symptoms were associated with Internet use for health information (Table 2). Women who accessed preventive blood pressure and skin cancer checks or who had ever had a live birth or pregnancy loss were less likely to use the Internet for health information.

**Table 1:** Sociodemographic characteristics of women who do and do not use the Internet as a health information resource (N=17,069).

Sociodemographics		Internet	Non-Internet	Internet use	
		n=7433	n=9636	OR (95% CI)	AOR^a^ (95% CI)
Age (years), mean (SD)		20.7 (1.67)	20.4 (1.69)	1.10 (1.08-1.12)	1.08 (1.06-1.11)
**Education, n (%)**					
	<Year 12	477 (6.43)	794 (8.39)	0.79 (0.70-0.89)	0.79 (0.69-0.89)
	Year 12	3170 (42.70)	4171 (44.09)	1	1
	Certificate/diploma	1808 (24.35)	2620 (27.70)	0.91 (0.84-0.98)	0.83 (0.77-0.90)
	University	1969 (26.52)	1875 (19.82)	1.38 (1.28-1.49)	1.17 (1.07-1.27)
**Area of residence, n (%)**					
	Major city	5773 (77.91)	7076 (73.68)	1	1
	Inner regional	1114 (15.03)	1717 (17.88)	0.80 (0.73-0.86)	0.82 (0.76-0.90)
	Outer regional	444 (5.99)	707 (7.36)	0.77 (0.68-0.87)	0.79 (0.70-0.90)
	Remote/very remote	79 (1.07)	104 (1.08)	0.93 (0.69-1.25)	0.96 (0.71-1.29)
**Marital status, n (%)**					
	Never married-single	2771 (37.32)	3733 (39.46)	1	1
	Never married-in a relationship	4031 (54.30)	4913 (51.93)	1.11 (1.04-1.18)	1.09 (1.02-1.16)
	Engaged/married	558 (7.52)	744 (7.86)	1.01 (0.90-1.14)	0.96 (0.85-1.09)
	Separated/divorced/other	64 (0.86)	70 (0.74)	1.23 (0.87-1.73)	1.31 (0.92-1.85)
**Ability to manage on income, n (%)**					
	Easy/not bad	2955 (39.82	3623 (38.31)	1	1
	Difficult some of the time	2619 (35.29)	3403 (35.98)	0.94 (0.88-1.01)	0.95 (0.87-1.02)
	Difficult all of the time/impossible	1847 (24.89)	2432 (25.71)	0.93 (0.86-1.01)	0.98 (0.91-1.07)
**Living arrangements, n (%)**					
	Living with parents	3639 (49.04)	4843 (51.21)	1	1
	Not living with parents	3782 (50.96)	4615 (48.79)	1.09 (1.03-1.16)	1.05 (0.98-1.12)

^a^ Mutually adjusted for other variables in the model.

**Table 2:** The association between physical, mental, and reproductive health and using the Internet as a health information resource (N=17,069).

Health-related variables		Internet, n (%)	Non-Internet, n (%)	Internet use	
		n=7433	n=9636	OR (95% CI)	AOR^a^ (95% CI)
**Physical health**					
	Self-rated general health				
	Excellent/very good	3191 (42.93)	3987 (42.10)	1	1
	Good	2982 (40.12)	3884 (41.01)	0.96 (0.90-1.03)	1.02 (0.95-1.09)
	Fair/poor	1260 (16.95)	1599 (16.88)	0.98 (0.90-1.07)	1.08 (0.99-1.18)
**Chronic condition**					
	None	3496 (47.04)	4425 (46.73)	1	1
	1	2673 (35.97)	3407 (35.981)	0.99 (0.93-1.06)	0.99 (0.93-1.06)
	≥2	1263 (16.99)	1638 (17.30)	0.97 (0.90-1.06)	0.97 (0.89-1.06)
**Urinary/bowel symptoms**					
	Never/rarely	3782 (50.89)	5656 (59.73)	1	1
	Sometimes/often	3650 (49.11)	3814 (40.27)	1.43 (1.35-1.52)	1.44 (1.36-1.54)
**Blood pressure check**					
	No	1096 (14.75)	1288 (13.61)	1	1
	Yes	6332 (85.25)	8177 (86.39)	0.91 (0.83-0.99)	0.85 (0.78-0.93)
**Skin cancer check**					
	No	5297 (71.32)	6608 (69.87)	1	1
	Yes	2130 (28.68)	2850 (30.13)	0.93 (0.87-1.00)	0.90 (0.84-0.97)
**Mental health**					
	Psychological distress				
	Low	1451 (19.54)	2078 (21.95)	1	1
	Moderate	2178 (29.33)	2814 (29.72)	1.11 (1.02-1.21)	1.13 (1.04-1.24)
	High	2173 (29.26)	2458 (25.96)	1.27 (1.16-1.38)	1.34 (1.23-1.47)
	Very high	1625 (21.88)	2118 (22.37)	1.10 (1.00-1.21)	1.24 (1.13-1.37)
**Diagnosed mental health condition**					
	No	4188 (56.36)	5559 (58.70)	1	1
	Yes	3243 (43.64)	3911 (41.30)	1.10 (1.04-1.17)	1.16 (1.09-1.23)
**Reproductive health**					
	Live birth				
	No	7063 (95.20)	8835 (93.45)	1	1
	Yes	356 (4.80)	619 (6.55)	0.72 (0.63-0.82)	0.74 (0.64-0.86)
**Pregnancy loss or termination**					
	No	6777 (91.30)	8516 (90.01)	1	1
	Yes	646 (8.70)	945 (9.99)	0.86 (0.77-0.95)	0.88 (0.79-0.98)
**Endometriosis**					
	No	7196 (96.81)	9295 (96.46)	1	1
	Yes	237 (3.19)	341 (3.54)	0.90 (0.76-1.06)	0.89 (0.75-1.05)
**Polycystic ovary syndrome**					
	No	7023 (94.48)	9082 (94.25)	1	1
	Yes	410 (5.52)	554 (5.75)	0.96 (0.84-1.09)	0.93 (0.82-1.07)
**Sexually transmitted infection**					
	No	6549 (88.13)	8420 (88.91)	1	1
	Yes	882 (11.87)	1050 (11.09)	1.08 (0.98-1.19)	1.06 (0.96-1.16)
**Menstrual symptoms**					
	Never/rarely	1165 (15.67)	1729 (18.26)	1	1
	Sometimes/often	6268 (84.33)	7742 (81.74)	1.20 (1.11-1.30)	1.25 (1.15-1.36)
**Pap test**					
	No	3796 (51.11)	4990 (52.72)	1	1
	Yes	3631 (48.89)	4475 (47.28)	1.07 (1.00-1.13)	0.94 (0.88-1.01)

^a^ Adjusted for age, education, area of residence, and marital status.

## Discussion

This study describes the sources of health information accessed by young Australian women and identifies the sociodemographic, physical, mental, and reproductive health factors associated with searching the Internet for health information. Our findings suggest that although the majority of young Australian women rely on their doctor for health information, a large proportion (43.55%, 7433/17,069) also access health information online. Several other studies, including a previous survey of Australian women across a wide age range [9], reported that although doctors are rated as the preferred and most credible source of health information, the Internet is another common source [5,8,20]. There is evidence to suggest that between 40% and 66% of adults use the Internet for health information [1-3,5,11]. Our finding that 44% of women aged 18-23 years used the Internet as a source of health information is generally consistent with previous estimates, although slightly lower (48.5%) than a recent large study of young French adults [1].

Consistent with several other studies [1,4,6-8], there were sociodemographic differences between those who did and did not use the Internet as a source of health information. Although the association between age and Internet use for health is conflicting, Internet use increased with age in our study among young women in the age range of 18-23 years. Young women’s preferences for online health information may increase in response to major life transitions and events that influence their health and well-being, including sexual and reproductive issues and events. Further, like other studies reporting a positive association between online health seeking and socioeconomic position [1,6,8], we found that young women who had a university qualification were more likely to search the Internet for health information. Women with higher educational qualifications are likely to have greater access to computers and the Internet and it is also possible that they find it easier to navigate the diversity of information offered by the Internet [20-22].

Women reporting “stigmatized” conditions or symptoms were more likely to search the Internet for health information. Consistent with other studies [1,10], we found that psychological distress and a diagnosis of a mental health condition were associated with Internet use. The stigma associated with mental illness is a common barrier to young adults’ use of professional support services [23]; however, the Internet may be an acceptable “flexible” resource that can offer “anonymized” information or support [24,25]. In Australia, several government-supported organizations including “headspace,” “beyondblue,” and “Young and Well” offer online resources to people with mental health issues. It is possible that some young women in our study accessed these websites independently or were advised by a health professional. Further, young women experiencing urinary and/or bowel or menstrual symptoms—where discussions with health care professionals may be perceived as embarrassing—were more likely to use the Internet as a health information resource. A review of UK research regarding young adults’ health care needs and preferences also described accessibility and confidentiality as important aspects of health care [13]. Thus, the Internet may play an important role in supporting young women with “sensitive” health issues.

Few studies have examined the relationship between health status and searching the Internet for health information and the evidence is somewhat inconsistent. In our study, we found that women with children, those who had experienced pregnancy losses, and those accessing preventive health services were least likely to use the Internet. These are all women who are likely to be in contact with health care professionals, so their need for health information may already be met. In contrast, we found no association between self-rated general health or chronic conditions and Internet use for health information. Although other studies have reported that use of computer-based resources or online support groups are associated with more visits to a health care professional [26] or poor self-rated general health [7], more recent studies have also found no association between Internet use for health and self-reported general health [1,6], chronic conditions [1], or number of visits to a health professional [3,6].

Although we assessed women’s health status, we did not ask women about recent visits to a health care professional. Therefore, we cannot determine the impact of the Internet on health care use. Further, although we focused on women’s use of information for their own health, other studies suggest that some people will use the Internet to seek information for another’s health. This may be an important avenue for future research with young women as they transition through adulthood, particularly motherhood.

Internet availability and use has increased dramatically in Western countries in the last decade. Our findings suggest that the Internet may be an acceptable resource for young women experiencing stigmatized or sensitive health issues, which has important implications for the effectiveness of professionally supported self-care programs [27]. Although the Internet has great capacity as a health resource, the quality of the information offered varies considerably, and misinformation has the potential to negatively impact a person’s health and well-being. Therefore, a better understanding of young women’s online behaviors is important for developing strategies to assist and direct women to credible online health resources.

## Abbreviations

ALSWH: Australian Longitudinal Study on Women’s Health
AIDS: acquired immune deficiency syndrome
HIV: human immunodeficiency virus
STI: sexually transmitted infection
TAFE: Technical and Further Education
